# The management of retroperitoneal giant schwannomas in AIDS patients: A case report

**DOI:** 10.3892/ol.2013.1191

**Published:** 2013-02-13

**Authors:** ZHANG KE, MU YI, JIANG LI, HUANG-RONG HAI, LU YAN, HE RONG, DING-ZHEN HAO, GUO-LI MIN, LI-QIN TAO, LI-BAO LIANG, JIA ZHE

**Affiliations:** Department of Hepatobiliary Surgery, Beijing Ditan Hospital, Capital Medical University, Beijing 100015, P.R. China

**Keywords:** acquired immunodeficiency syndrome, surgery, retroperitoneal schwannoma

## Abstract

Retroperitoneal schwannomas are a rare disease. During the potent antiretroviral therapy era, the incidence of AIDS-defining cancers has decreased, while the incidence of non-AIDS defining cancers has increased; however, the existence of a relationship between benign or malignant schwannomas and AIDS remains unclear. Although a case of ethmoid malignant schwannoma in an AIDS patient was first reported in 1993, no additional reports of schwannomas associated with AIDS have been published since. In the current study, the case of a 30-year-old male AIDS patient with a large benign retroperitoneal schwannoma is presented. The ideal treatment of retroperitoneal schwannomas is complete excision. However, controversy exists over the necessity of negative soft tissue margins, particularly when adjacent tissue or viscera must also be removed. In the current case study, due to the immune dysfunction in AIDS patients, the incidence of malignancy could not be completely excluded prior to surgery and a significant risk of short-term relapse or malignancy following partial tumor resection was present. The patient underwent complete resection with partial superior mesenteric artery excision in order to attain negative margins, and recovered well. A follow-up was performed 1 year after the procedure and the patient was well and a CT scan demonstrated no evidence of recurrence. However, the long term efficacy of this procedure requires continued observation in this patient.

## Introduction

Nerve sheath tumors are a subclass of soft-tissue neoplasms that include benign and malignant schwannomas and neurofibromas. Schwannomas may occur in any organ or nerve trunk, with the exception of cranial nerves I and II, which lack Schwann cells. Schwannomas are associated with von Recklinghausen disease in 5–18% of cases. In the absence of von Recklinghausen disease, these masses rarely occur in the retroperitoneum, and comprise just 3% of all schwannomas (malignant and benign combined). Retroperitoneal schwannomas are usually larger and have a higher tendency to undergo spontaneous degeneration and hemorrhage (1).

During the potent antiretroviral therapy era, the incidence of AIDS-defining cancers (ADCs) has decreased and the incidence of non-AIDS-defining cancers (NADCs) has increased. This increase in NADCs is partly a consequence of increased immune activation and decreased immune surveillance as well as direct effects of HIV (2). Although Trimas *et al* first described the clinical presentation of a malignant schwannoma arising from the ethmoid sinus in an AIDS patient in 1993 (3), no additional studies on schwannomas associated with AIDS have followed. The relationship between benign or malignant schwannomas and AIDS remains undefined. To the best of our knowledge, this is the first description of benign retroperitoneal schwannomas in an AIDS patient cured with surgery. Written informed consent was obtained from the patient.

## Case report

**Figure 1:**
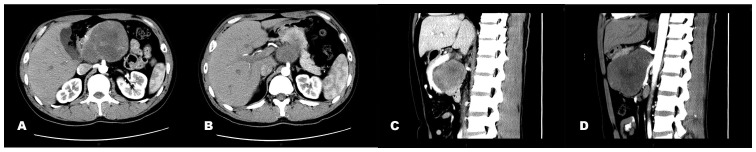
CT scan presents a 10-cm heterogeneous retroperitoneal solid mass on the (A) abdominal aorta and (B) closely attached to the posterior wall of the pancreas. The mass is surrounded by the (C) portal vein, (D) celiac trunk and superior mesenteric artery.

A 30-year-old male with a 1-year history of asymptomatic AIDS presented with mild 6-month abdominal discomfort. Upon physical examination, an evident mass was identified in the upper abdomen. CT scan revealed a 10-cm heterogeneous retroperitoneal solid mass located on the abdominal aorta and closely attached to the posterior wall of the pancreas (Fig. 1A and B). The mass was surrounded by major abdominal blood vessels, including the portal vein and its major tributary, celiac trunk and its major branches as well as the superior mesenteric artery (Fig. 1C and D). Laboratory analysis revealed that CD4^+^ T lymphocyte counts were 561 cells/*μ*l and the ratio of CD4^+^/CD8^+^ was 1.03. An infectious disease specialist recommended that it was not necessary to administer antiretroviral therapy prior to surgery. An exploratory laparotomy was performed using a midline incision. The large retroperitoneal tumor was found in the portacaval gap and displacing corpus pancreatis and C-loop of the duodenum. The tumor was surrounded by the major abdominal blood vessels consistent with the CT scan results and exhibited marked neovascularity arising from the splenic and superior mesenteric vein. In addition, the tumor had undergone tight adhesion with the superior mesenteric artery. *En bloc* resection of the tumor was performed by excising a 1-cm length section of the superior mesenteric artery, followed by reconstruction of the artery by end-to-end vascular anastomosis. Upon microscopic examination, benign Schwannian cells, along with an alternating Antoni A and Antoni B pattern and areas of nuclear atypical were noted. The specimen tested positive for S100. The patient had an uncomplicated recovery and was treated with standard antiretroviral treatment on postoperative day 14. During the 1-year follow-up period, the patient was disease-free.

## Discussion

Schwannomas are usually benign and are associated with von Recklinghausen disease in 5–18% of cases. Malignancy is extremely rare and is usually observed in patients with von Recklinghausen disease. Of all benign schwannomas, only 0.3–3.2% are retroperitoneal. Retroperitoneal schwannomas are generally solid encapsulated tumors that arise from the paravertebral region. These are usually asymptomatic and are hypothesized to grow slowly. The tumors frequently attain a relatively large size prior to identification due to the flexible and nonrestrictive properties of the retroperitoneum (4). Retroperitoneal schwannomas are commonly detected preoperatively via cross-sectional imaging. However, preoperative diagnosis is difficult as none of these modalities have revealed any pathognomonic features unique to this tumor. The CT scan is more useful for the detection of specific characteristics of the tumor. Size, exact location, relationship with other organs and invasion are accurately reproduced (5). In the present study, a CT scan clearly demonstrated the anatomical relationship between the tumor and the abdominal organs or important abdominal blood vessels and was extremely useful for determination of surgical risk and procedure design.

The incidence of NADCs has increased >3-fold over the last 10 years and has now surpassed that of ADCs in HIV-infected patients. Among types of NADCs, incidences of prostate and breast cancer have remained relatively constant and those of cervical, anal and lung cancer have increased. Contributing factors to the increased prevalence of NADCs include: i) greater prevalence of coinfection with viruses that have etiological roles in cancer; ii) behaviors and environmental toxins; and iii) effects of HIV infection, including potential direct effects of the virus and the consequences of long-term immunosuppression. Previous studies have indicated that HIV-infected cancer patients have a poorer prognosis than similarly staged non-HIV-infected patients with the same cancer. Although these studies also report that HIV-infected cancer patients should receive at least the same intensity of cancer therapy as non-HIV-infected patients, a considerable lack of understanding still exists with regards to the treatment and management of patients with NADCs (6).

The ideal treatment of retroperitoneal schwannomas is complete excision. However, controversy exists over the necessity of negative soft-tissue margins, particularly when adjacent tissue or viscera must also be removed. Although specific investigators advocate complete excision, including the sacrifice of adjacent tissue if necessary, others indicate that due to the benign nature of this tumor, simple enucleation or partial excision is sufficient (7). In the current case study, due to the immune dysfunction in AIDS patients, the incidence of malignancy could not be completely excluded prior to surgery and a significant risk of short-term relapse or malignancy following partial tumor resection was present. The patient underwent complete resection with partial superior mesenteric artery excision in order to attain negative margins, and recovered well. A follow-up was performed 1 year after the procedure and the patient was well and a CT scan demonstrated no evidence of recurrence. However, the long-term efficacy of this procedure requires continued observation in this patient.

In order to reduce surgical trauma, postoperative complications and the risk of intraoperative cross-infection with HIV, laparoscopic resection of retroperitoneal schwannomas is more suitable compared to open laparotomy for use in AIDS patients. Laparoscopic resection has also been used to treat retroperitoneal schwannomas in non-HIV-infected patients. Hemorrhage is a serious intraoperative risk in cases where major vessels are situated near to the tumor, and there are currently several reports of unsuccessful tumor excision and intraoperative mortalities (8). Minimally invasive robotic surgery has been used in various surgical procedures due to its high accuracy, fine manipulation capability and high reliability. We have implemented several cases of cholecystectomy and hepatectomy in AIDS patients using the Da Vinci surgical robot system; however, experience in robotic surgery, particularly for complex abdominal surgical procedures, remains insufficient. The application of this technology in surgery on AIDS patients requires ongoing development.
